# Supplementary material for: Octave-spanning Kerr soliton frequency combs in dispersion- and dissipation-engineered lithium niobate microresonators
Source: Light Sci Appl. 2024 Sep 2;13:225. doi: 10.1038/s41377-024-01546-7 (PMC11369083; doi:10.1038/s41377-024-01546-7)
Supplement: Supplementary file 1 — Supplementary Information [file 41377_2024_1546_MOESM1_ESM.pdf]

Supplementary Information for: Octave-spanning Kerr soliton frequency combs in dispersion- and dissipation-engineered lithium niobate microresonators

Yunxiang Song[1,2,*], Yaowen Hu[1], Xinrui Zhu[1], Kiyoul Yang[1,*], Marko Lončar[1,*]

[1]John A. Paulson School of Engineering and Applied Sciences, Harvard University, Cambridge, MA 02138, USA.

[2]Quantum Science and Engineering, Harvard University, Cambridge, MA 02138, USA.

[*]Corresponding authors: ysong1@g.harvard.edu, kiyoul@seas.harvard.edu, loncar@g.harvard.edu

I. DISSIPATIVE KERR SOLITON DEVICE DESIGN GUIDELINES

In the main text, we proposed free spectral range (FSR) control and dissipation engineering methods for dissipative Kerr soliton (DKS) generation from thin-film lithium niobate (TFLN) microrings, instead of stimulated Raman scattering (SRS) of the pump light. Both methods are based on reducing the ratio between FWM and SRS thresholds below unity, where the ratio is defined by

$$\zeta = \frac{P_{\text{th}}^{\text{Kerr}}}{P_{\text{th}}^{\text{SRS}}} \sim \frac{Q_L^R}{Q_L^P} \cdot \frac{g_{\text{eff}}^{\text{SRS}}(\delta)}{g^{\text{Kerr}}} < 31 \cdot \frac{Q_L^R}{Q_L^P}. \tag{1}$$

Here, $P_{\text{th}}^{\text{Kerr}}, P_{\text{th}}^{\text{SRS}}$ are the FWM and SRS power thresholds, Q_L^R, Q_L^P are the loaded quality factors of the Raman mode and pump mode, g^{Kerr} is the FWM gain coefficient, $g_{\text{eff}}^{\text{SRS}}(\delta)$ is the Raman gain function, and $\delta = f^R - (f^P - \Delta)$ is the detuning between the Raman mode and the Raman gain peak $f^P - \Delta$. In Z-TFLN, the fundamental TE mode couples to the E(LO_8) vibrational mode characterized by a Raman shift of $\Delta \sim 18.94$ THz and a Raman gain bandwidth of $\Gamma \sim 558$ GHz[1,2]. The factor of 31 arises from the ratio between SRS peak gain (evaluated at $\delta = 0$) and the C-band four-wave-mixing gain (Methods), where f^P, f^R are the pump and Raman mode frequencies. In the FSR control method we utilized point-coupled, large FSR microrings and the placement of microring modes symmetrically about the Raman gain peak. However, this method places very stringent requirements on the FSR, such as disallowing certain bands of FSRs and setting FSR lower bounds. On the other hand, the dissipation engineering method does not require specific FSRs nor imposes lower bounds on the FSR, and near-unity DKS yield is maintained across multiple FSRs and microring radii. In the following, we discuss the design of pulley couplers for SRS suppression, explore Q_c trends with pulley coupler parameters, and offer physical intuitions for such trends.

Device parameters for dissipation-engineered microrings are shown in Fig. S1. Assuming a critically coupled pump mode and a broadband intrinsic quality factor (Q_i) of 1 million, pulley-coupled microrings feature $Q_c^R/Q_c^P \lesssim 1/40$ when the coupling resonance is just higher frequency than the C-band. On the other hand, point coupled microrings provide only $Q_c^R/Q_c^P \sim 1/5$, as shown in Fig. S2. The small Q_c^R/Q_c^P renders dissipation engineering, which utilizes high Q_c contrast near a coupling resonance, as effective in lowering ζ below unity.

Precise tuning of the coupling resonance location, such that it is just higher frequency than the C-band, while the pump remains critically coupled, is central to the dissipation engineering method. In Fig. S3, we explore the pulley coupler parameter space and the effects of each parameter on the frequency-dependent Q_c retrieved from finite-difference time-domain (FDTD) simulations (Methods). Prior to interpreting 3-D simulation results, we introduce the coupled-mode equations to describe this system and their consequences[3]. The purpose is to interpret simulation results in a physical manner, leveraging the five relationships stated in Moille et al.:

$$\Gamma(f) = \frac{j\pi f}{2} \int_{A_{\text{bus}}} (\epsilon_{\text{bus}} - \epsilon_{\text{ring}}) \cdot E_{\text{ring}}^*(f) \cdot E_{\text{bus}}(f) \cdot drdz, \tag{2}$$

$$\phi = l\sqrt{(\Delta\beta/2) + \Gamma^2(f)}, \tag{3}$$

$$t(f) = \Gamma(f) \int_L e^{j\phi} dl, \tag{4}$$

$$\begin{aligned} t(f) &= 2\Gamma(f) \cdot \frac{\sin\left(\frac{L}{2}\sqrt{(\Delta\beta)^2 + \Gamma^2(f)}\right)}{\sqrt{(\Delta\beta)^2 + \Gamma^2(f)}} \\ &= \Gamma(f) \cdot L \cdot \text{sinc}\left(\frac{L}{2}\sqrt{(\Delta\beta)^2 + \Gamma^2(f)}\right), \end{aligned} \tag{5}$$

$$Q_c(f) = \frac{2\pi f}{|t(f)|^2 \cdot \text{FSR}}. \tag{6}$$

Here, f indicates frequency dependence, Γ is the bus waveguide to microring waveguide mode overlap factor, $\epsilon_{\text{bus}}, \epsilon_{\text{ring}}$ the dielectric permittivity distribution when only the bus or microring waveguide is present, $E_{\text{ring}}, E_{\text{bus}}$ the electric field distribution of the bus and waveguide modes, A_{bus} the bus waveguide area to be integrated over, ϕ the phase mismatch between bus and microring waveguide modes accumulated throughout the pulley coupler, $\Delta\beta$ the wavevector mismatch between bus and waveguide modes, t the complex transmission of the bus to microring waveguide per-pass, FSR the free spectral range of the microring, and, finally, Q_c the

frequency-dependent coupling quality factor (dissipation spectrum). Note that such equations are capable of constructing $Q_c(f)$ curves for dissipation engineering simulation, only requiring independent 2-D mode simulations of E_{ring} and E_{bus} which is computationally simpler than 3-D FDTD simulations. However, we found that $Q_c(f)$ resulting from a 2-D simulation typically infers less coupling than that from a full 3-D simulation of a coupler with identical parameters. This is likely due to finite residual coupling introduced by two regions in the vicinity of the uniform coupling gap, as the bus waveguide diverges away from the microring waveguide, which is not accounted for in coupled-mode equations as written above. Calibrated adjustments to the interaction length (coupling angle) may be applied to account for the neglected residual coupling, either through numerically computing an effective additional length with a number of 2-D mode simulations along the diverging-gap region[3], or through sufficient iteration looking at the differences between constant-gap simulations and measurement. Our intention here for describing the coupled-mode equations[3] is to provide enough physical intuition so as to permit physics-informed 3-D simulation optimization instead of extensive parameter sweeping which can be computationally intensive and time consuming.

First, we consider the bus waveguide width B. This parameter affects Q_c by changing the $\Delta\beta$ and $\Gamma(f)$. Since $\Delta\beta$ is proportional to the effective index difference between the bus and microring waveguide modes, $n_{\text{eff, bus}}$ responds strongly to small changes in B due to the single-mode nature of the bus waveguide. In the single-mode limit, refractive index contrast between core (Z-TFLN) and top cladding (air) is sensitive to B, and this contribution reflects in Eq. (2) and (3). However, a wider bus width also shifts the bus mode away from the microring mode, decreasing the overlap integral in $\Gamma(f)$. The overall effect on Q_c is shown in Fig. S3a, where we observe that an increase in B from 0.8 to 0.95 μm (all other parameters fixed) decreases overall t, raises Q_c slightly, and frequency downshifts the coupling resonance. Importantly, small variations in B can flexibly tune the position of the coupling resonance and is especially useful for positioning the pump mode in a position of high Q_c sensitivity to frequency.

Second, we consider the coupling gap G. Intuitively, a larger gap decreases the overlap between bus and microring waveguide modes which decreases $\Gamma(f)$. As shown in Fig. S3b, while larger gaps can place coupling resonances at desired locations, the Q_c curve may be raised by or lowered by orders of magnitude when G is increased from 1.0 to 1.3 μm (all other parameters fixed). This presents difficulty for (i) achieving a critically coupled pump near a coupling resonance and (ii) extracting higher frequency (shorter wavelength) components of a broadband DKS. Therefore, it is important to converge on suitable G such that the overall Q_c reasonably extracts light over the entire DKS bandwidth, and then sweep B to re-position the coupling resonance just higher frequency than the pump mode.

Third, we consider the interaction angle A, defined as $A = 360^\circ \cdot (L/2\pi R)$, where L is the pulley length and R is the microring radius. When A is varied from 30° to 60° (all other parameters fixed), the $t(f)$ changes due to an increase in L: its absolute magnitude for a fixed frequency increases due to the linear dependence on L, and its period is shortened due to L by the sinc($\cdot$) function. As shown in Fig. S3c, Q_c is slightly lowered suggesting increased coupling, and more than one coupling resonance emerges within the simulation bandwidth. Therefore, A not only provides a tuning knob to position pump modes near a coupling resonance, but also significantly lowers Q_c in the range of frequencies between coupling resonances. This feature further enables spectrally-tailored, enhanced extraction of comb power in the higher frequency (lower wavelength) regime, where outcoupling is generally weak due to small $\Gamma(f)$.

Finally, we consider the microring width W. We find that W has little effect on Q_c, as shown in Fig. S3d. This is expected due to the multi-mode nature of the microring waveguide. Small changes in W do not significantly affect the refractive index composition (of core and cladding) which the fundamental transverse electric mode experiences, leading to only small changes on $n_{\text{eff, ring}}$ and thus $t(f)$. This is convenient since W is one of the central tuning knobs for dispersion engineering the DKS span. Our results show that dispersion engineering using W mostly decouples from dissipation engineering and suggests broadband DKS generation and SRS suppression are not mutually exclusive.

In practice, we fabricate devices around simulated optimal points, since our fabrication process involving iterative wet etching tends to cause deviations from initial design. For example, the dissipation-engineered, 50 μm-radius microring in Fig. 1 of the main text exhibited measured Q_c different from its simulation using as-designed parameters. However, increasing the coupling gap (G) by 60 nm and the waveguide height (H) by 2 nm, well within fabrication tolerances of our process, matched simulated Q_c with the measured Q_c sampled within the C/L-band pump bandwidth (184.0-198.7 THz range). This comparison is shown in Fig. S4 and illustrates the sensitivity of dissipation engineering to parameter combinations. The requirement of positioning the coupling resonance just higher frequency than the C-band while critically coupling the C-band, can be rather sensitive to small variations in B, A, G, and H, but not W.

II. DISSIPATIVE KERR SOLITON YIELD CHARACTERIZATION

The DKS yield is determined by screening through a large number of microrings fabricated following the FSR control or dissipation engineering methods. The screening process involves sweeping a sufficiently powerful pump (about 100 mW on-chip power) back and forth

across microring resonance modes in the C-band. This is schematically shown in Fig. S5a. The output light passes through a fiber Bragg grating (FBG) tuned to reflect the pump frequency and the FBG pass band is collected by a 125 MHz photodetector. The slow electrical signal is displayed on an oscilloscope. Oscilloscope signatures of SRS (DKS) generation, for both directions of the pump frequency scan, are shown in Fig. S5b, c (S5d, e). We note that the SRS trace produces very low photodetector voltage (inefficient SRS), which is likely due to local variation in loaded quality factor raising the DKS threshold despite a strongly suppressed SRS. Otherwise, when SRS is completely suppressed, the full space of DKS states are revealed in the blue to red scan, while a subset of DKS states are directly accessed and modulation instabilities are revealed in the red to blue scan. This distinction is a result of photorefraction-induced microring resonance blue shift creating various transition pathways of the intracavity state into DKS resonance branches[4]. Such a distinction does not present any limitations operationally, since manual tuning into the desired DKS state is always possible, in at least one direction. We note that blue-to-red manual tuning is usually utilized in this work for initiating free-running DKS states and subsequent comb spectra collection. In the DKS yield statistics, microring resonance modes generating oscilloscope traces like Fig. S5b, c contribute to an SRS count and those resembling discrete DKS steps, as in Fig. S5d, e, contribute to a DKS count. In Fig. S6, we provide 32 comb power traces collected over resonance modes of eight dissipation-engineered microrings, with radii in the 40-60 μm range.

III. EXPERIMENTAL DETAILS AND FURTHER IMPROVEMENTS

Experimental setups for collection of oscilloscope traces (DKS comb power and SRS signatures) and comb spectra are shown in the top and bottom panels of Fig. S7, respectively.

In the main text, we discussed improved loaded quality factor using fully etched ridge waveguides, coupled with increased anomalous dispersion, as a further opportunity for improving the DKS comb span, such that dispersive waves are one octave apart. Fig. S8 (red curve) shows an integrated dispersion D_{int} for a near fully etched waveguide (470 nm waveguide height, 480 nm initial Z-TFLN thickness), which pushes the high frequency (low wavelength) dispersive wave significantly outward. The dispersive waves are located at approximately 137 THz and 273 THz. Compared to both octave-spanning devices in the main text (blue and green curves), microrings with such D_{int} have larger $D_2(f^P)$ and increased DKS threshold, which must then rely on dissipation engineering methods and the higher intrinsic quality factors of tightly-confining ridge waveguides[5] for the realization of octave-spanning DKS from them.

1. Basiev, T. *et al.* Raman spectroscopy of crystals for stimulated Raman scattering. *Optical materials* **11**, 307–314 (1999).
2. Ridah, A., Bourson, P., Fontana, M. & Malovichko, G. The composition dependence of the Raman spectrum and new assignment of the phonons in $LiNbO_3$. *Journal of Physics: Condensed Matter* **9**, 9687 (1997).
3. Moille, G. *et al.* Broadband resonator-waveguide coupling for efficient extraction of octave-spanning microcombs. *Optics letters* **44**, 4737–4740 (2019).
4. He, Y. *et al.* Self-starting bi-chromatic $LiNbO_3$ soliton microcomb. *Optica* **6**, 1138–1144 (2019).
5. Gao, Y. *et al.* Compact lithium niobate microring resonators in the ultrahigh Q/V regime. *Optics Letters* **48**, 3949–3952 (2023).

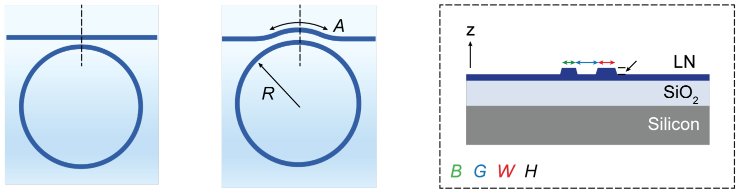

Fig. S1. **Device parameters.** Point coupled and pulley coupled microrings used in the FSR control and dissipation engineering methods. Device parameters in the top-down views and cross section of the coupling region (black dashed line) are labeled as follows. R: microring radius, A: interaction angle, B: bus waveguide width, G: microring to bus waveguide coupling gap, W: microring waveguide width, H: waveguide height.

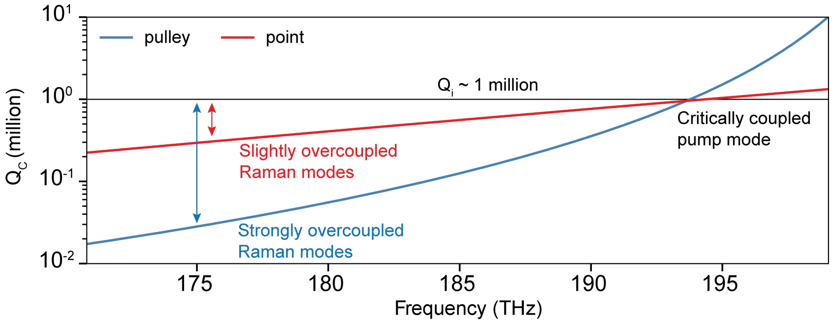

Fig. S2. **Comparison between point and pulley coupled microrings.** Finite-difference time-domain simulation of the coupling quality factor Q_c due to a point (red) and pulley (blue) coupled microring. Assuming an intrinsic quality factor of $Q_i \sim 1$ million (black horizontal line) throughout the plotted bandwidth and a critically coupled pump in the C-band ($Q_i^P \sim Q_c^P$), a pulley coupled microring yields $Q_c^R/Q_c^P \sim 1/40$ while a point coupled microring yields $Q_c^R/Q_c^P \sim 1/5$. The former case favors DKS generation over SRS regardless of the microresonator FSR, while the latter case cannot suppress SRS unless FSR control methods are utilized.

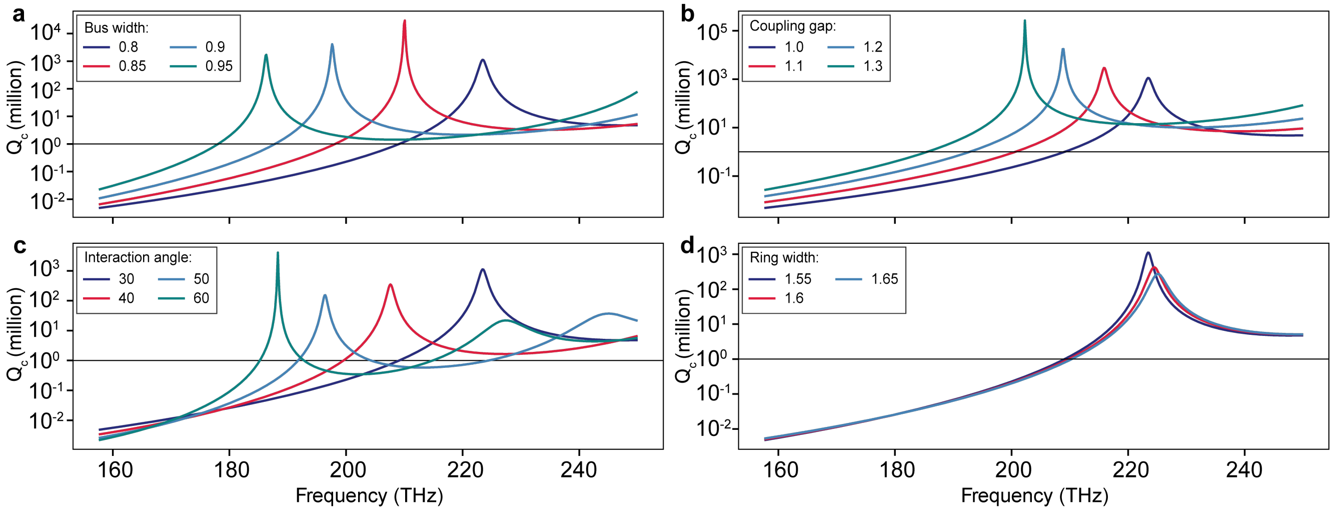

Fig. S3. **Coupling quality factor dependence on design parameters.** Q_c vs. microring mode frequency as a function of **a**, bus waveguide width, **b**, microring to bus waveguide coupling gap, **c**, interaction angle (length of pulley coupler as an angular fraction of the microring circumference), and **d**, ring waveguide width. Black horizontal line marks $Q_i \sim 1$ million, and its intersections with the Q_c curves mark frequencies of critical coupling ($Q_i \sim Q_c$).

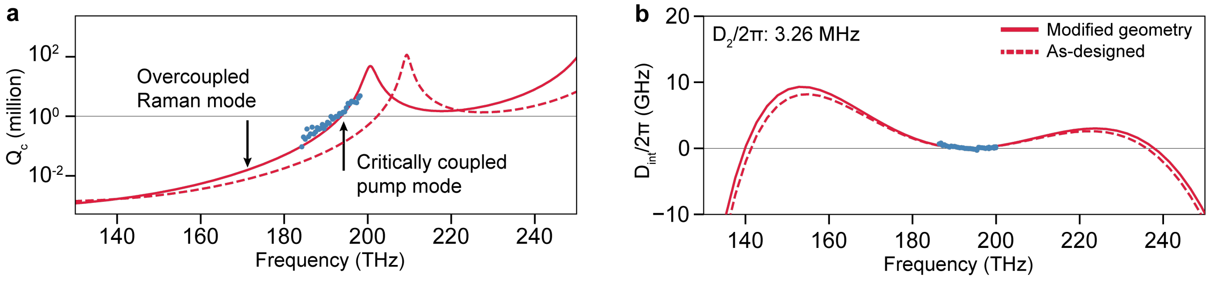

Fig. S4. **Coupling quality factor and integrated dispersion for designed vs. fabricated geometries.** **a**, Simulated coupling quality factor Q_c of the 50 μm-radius microring in Fig. 1 of the main text. Dashed red line is the simulation result based on parameters as-designed (bus waveguide width (B) 0.85 μm, microring waveguide width (W) 1.62 μm, coupling gap (G) 1.0 μm, interaction angle (A) 45°, and waveguide height (H) 0.328 μm). Blue dots are obtained by extracting Q_c from the microring resonances within the 184.0-198.7 THz frequency range. The deviation from the dashed red line is due to our fabrication process (Materials and Methods), where successive wet etches of Z-TFLN reduces fabricated dimensions from their initial designs. Accounting for this, we increased the coupling gap and etch depth, in simulation, to 1.06 μm and 0.33 μm, which yields the solid red line. This modified Q_c simulation matches well with measured Q_c and is in agreement with the coupling resonance probed by the outcoupled DKS spectra in Fig. 1e, f of the main text. Black line labels $Q_i \sim 1$ million. **b**, Simulated (solid and dashed red lines) and measured (blue dots) integrated dispersion D_{int} of the same 50 μm-radius microring. As-designed parameters and modified geometries yield nearly identical D_{int}. Black line labels $D_{\text{int}} = 0$.

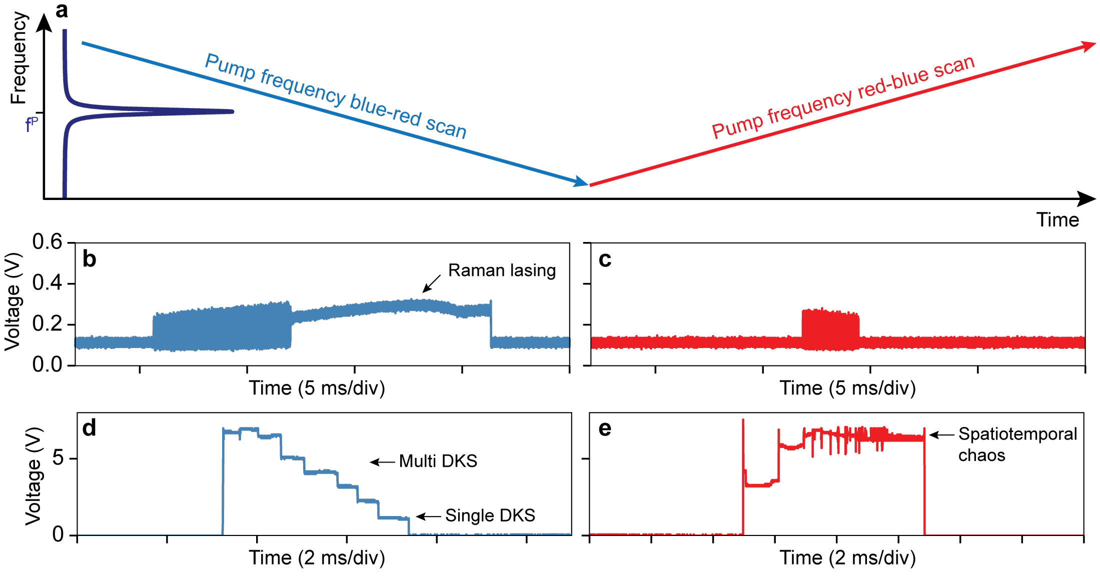

Fig. S5. **Comb power signatures of SRS and DKS.** **a**, Schematic of pump frequency sweep across a microring resonance mode at frequency f^P. The blue-to-red sweep direction is represented by blue lines and red-to-blue direction by red lines. Typical comb power trace (pump power removed using a fiber Bragg grating) as a photodetector voltage, for **b**, **c**, Raman lasing regime and **d**, **e**, DKS generation in the Raman suppressed, dissipation-engineered regime. In **d**, **e**, SRS is effectively suppressed over the entire range of DKS and Kerr-nonlinear frequency generation, evidenced by the absence of Raman lasing signatures.

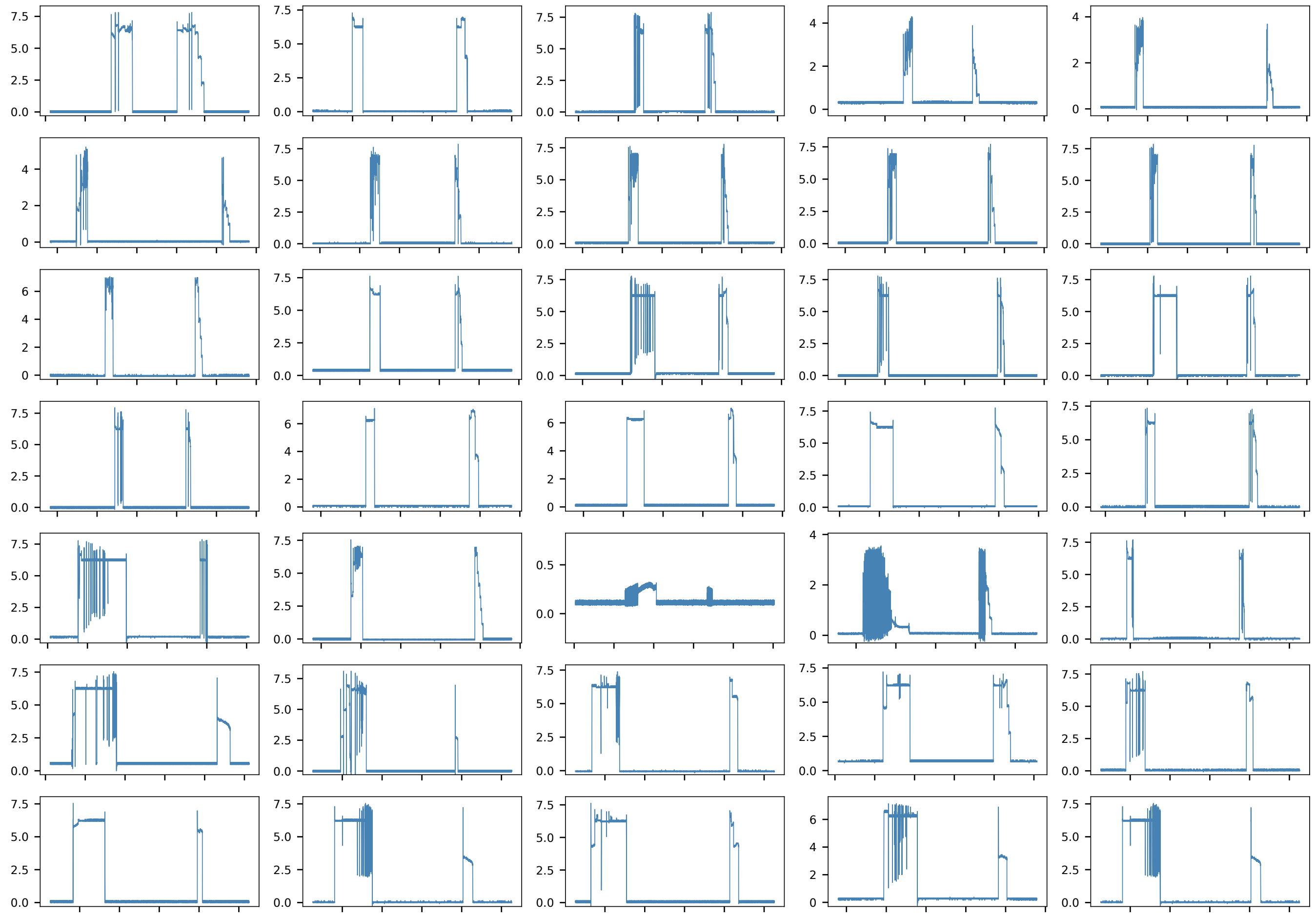

Fig. S6. **DKS yield determination.** Resonance modes across eight dissipation-engineered microrings, with radii in the 40-60 μm range, were pumped with 50-200 mW on-chip power and comb power traces recorded. Among these traces, 31 traces are in the DKS generation regime and one trace is in the Raman lasing regime (fifth row center plot, also enlarged in Fig. S3b, c). In all plots, vertical axis represents photodetector voltage and horizontal axis represents time.

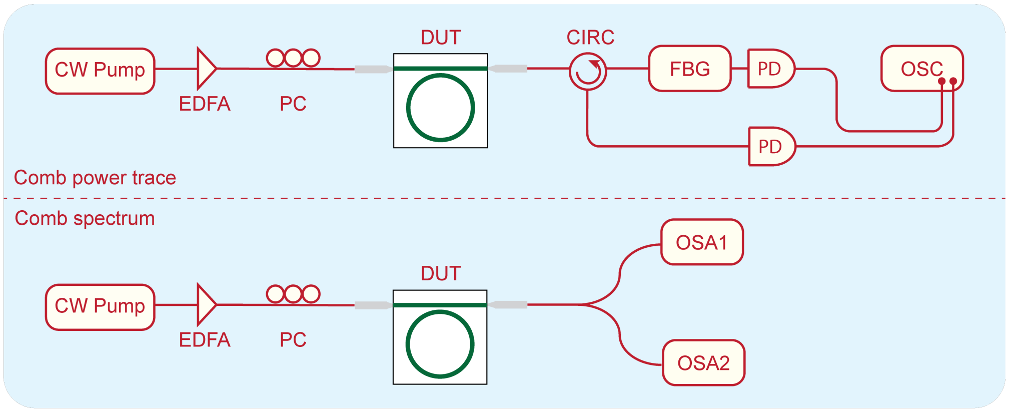

Fig. S7. **Experimental setups.** Top shows the experimental setup for collecting comb power traces, primarily used for rapid screening of DKS generation vs. SRS in the batch of microrings desgined based on the FSR control or dissipation engineering methods. Bottom shows the experimental setup for collecting comb spectra, in which case pump light is not removed using an optical circulator and fiber Bragg grating pair. Due to the frequency range of DKSs extending well below the 150 THz (above the 2 μm) level, SMF-28 fiber (silica) absorption of comb lines starts to play a role. Therefore, excess fiber length is minimized to preserve the DKS spectra and to accurately reflect its spectral content on-chip. Abbreviations in the figure are given as follows. CW: continuous wave, EDFA: erbium-doped fiber amplifier, PC: polarization control, DUT: device under test, CIRC: optical circulator, FBG: fiber Bragg grating, PD: photodetector, OSC: oscilloscope, OSA1(2): optical spectral analyzer 1200-2400 nm (600-1700 nm) model.

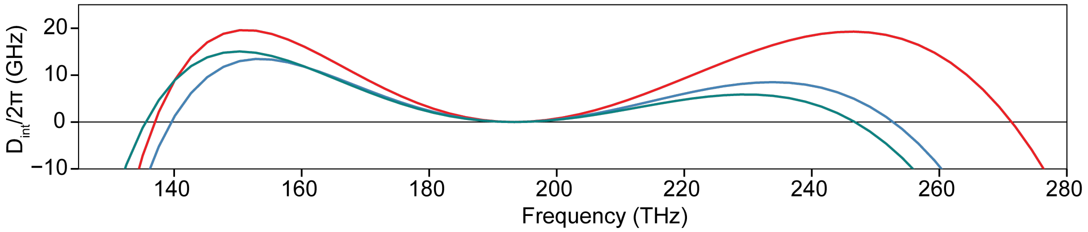

Fig. S8. **TFLN ridge waveguide integrated dispersion.** Simulated integrated dispersion D_{int} for a 470 nm etched waveguide starting from 480 nm initial Z-TFLN film thickness (red). Dispersive waves found near 137 and 273 THz are separated by one octave. Two octave-spanning D_{int}, corresponding to Fig. 4d, e of the main text, are plotted for comparison (blue, green).
